# A Rare Cause of Acute Abdomen: An Isolated Falciform Ligament Necrosis

**DOI:** 10.1155/2014/570751

**Published:** 2014-06-17

**Authors:** Ziya Taner Ozkececı, Mustafa Ozsoy, Bahadır Celep, Ahmet Bal, Coskun Polat

**Affiliations:** ^1^Department of General Surgery, Afyon Kocatepe University, Afyon, 1819/11 Sok. No. 16/10 İstasyonaltı Mahallesi, Çiğli, 35630 Izmir, Turkey; ^2^Department of General Surgery, Karabuk University, 78000 Karabuk, Turkey

## Abstract

The falciform ligament is one of the anatomical structures which attach the liver to the diaphragm and anterior abdominal wall. Primary falciform ligament is very rare. In this article, we present a case of an isolated falciform ligament necrosis, a rare primary pathology of the falciform ligament, who was admitted with acute abdomen. Case presentation: A 64-year-old female patient was admitted with the complaints of pain. Laboratory test results showed a leukocyte count of 17,000/mm^3^. Imaging studies demonstrated intra-abdominal reactionary fluid along with a heterogeneous mass localized in the falciform ligament. Exploratory laparotomy revealed a necrotic mass of the falciform ligament. No other pathology responsible for falciform ligament necrosis was found. We believe that falciform ligament necrosis should be considered a preliminary diagnosis, if any ligament abnormality, tumor, intraligament air density, or the presence of reactionary fluid surrounding the ligament is detected through abdominal imaging studies.

## 1. Introduction

The falciform ligament, which is located on the left of the midline of the abdomen, runs through the anterior wall of the abdomen and diaphragm. It is one of the anatomical structures which attaches the liver to the remnants of the umbilical veins. The length of the falciform ligament may vary individually and it contains the ligamentum teres and obliterated umbilical vein. It also contains paraumbilical veins. The falciform ligament artery originating from the liver was reported in several cases in the literature [1]. Furthermore, falciform ligament-related conditions are very rare. The ligament is usually secondarily affected by surrounding inflammatory diseases. However, there are only a few case reports of primary falciform ligament disease in the literature [2]. In this paper, we present a case that underwent urgent surgery due to acute abdomen, which was secondary to primary falciform ligament necrosis, in the light of literature data.

## 2. Case Report

**Figure 1:**
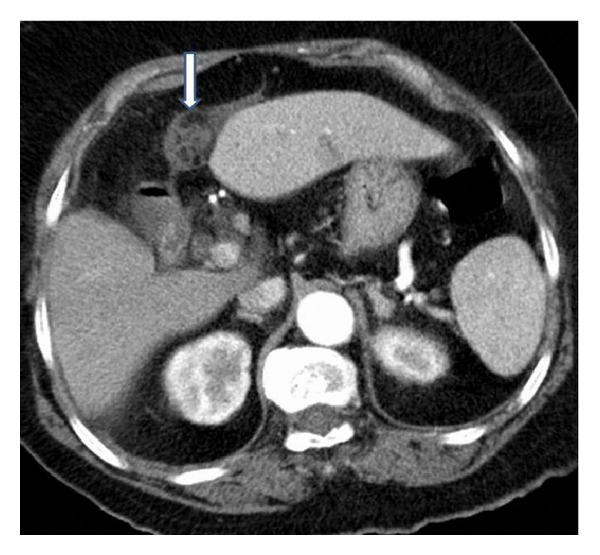
An abdominal computed tomography scan showing a mass lesion (arrow) originating from the gallbladder region extending to the periportal region and surrounding the falciform ligament with air density and reactionary fluid.

**Figure 2:**
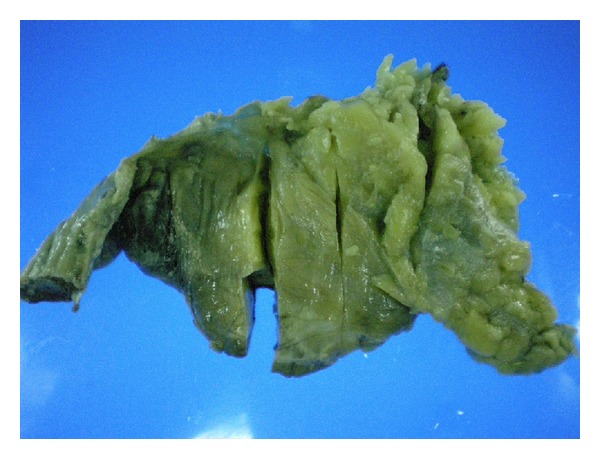
Macroscopic view of surgical specimens.

A 64-year-old female patient was admitted with the complaints of pain in the right upper quadrant and the epigastric region. The patient's history revealed an abdominal pain for 24 hours with an increasing severity, as well as nausea and vomiting. Physical examination suggested peritoneal irritation with a systolic blood pressure of 100/70 mmHg, pulse rate of 140 bpm, and body temperature of 38.5°C. Laboratory test results showed a leukocyte count of 17,000/mm^3^ (4000–10,000/mm^3^). Serum chemistry tests and electrolyte measurements did not indicate any pathology related to acute abdomen. In addition, CA19-9, CEA, CA-125, and AFP tumor biomarkers were normal. The air under diaphragm demonstrated was by posteroanterior X-ray, one of the radiographic diagnostic tools and also abdominal ultrasonography demonstrated intra-abdominal reactionary fluid along with a heterogeneous mass localized in the falciform ligament. Abdominal computed tomography showed a mass lesion originating from the gallbladder extending to the periportal region and surrounding the falciform ligament without opaque-medium enhancement (Figure 1). Based on these findings, the patient was immediately scheduled for laparotomy with the preliminary diagnoses of generalized peritonitis secondary to duodenal ulcer perforation or mesenchymal tumor perforation. Exploratory laparotomy revealed a necrotic mass of the falciform ligament with 1000cc reactionary fluid in the abdomen. The duodenum and other intestinal segments were normal. Intra-abdominal solid organs and gallbladder were also found to be normal. No other pathology responsible for falciform ligament necrosis was observed. The falciform ligament was resected. The histopathological examination of surgical specimens revealed a 10 × 13 × 4 cm falciform ligament necrosis with hemorrhagic regions, fibrin, and massive polymorphonuclear infiltration (Figure 2). The postoperative period was uneventful. A patient was discharged on the seventh postoperative day and also after 18-month follow-up, no complaints were found out.

## 3. Discussion

Although the anatomical structure and variations of the falciform ligament are definitely defined, associated conditions of the falciform ligament remain to be elucidated. The most common pathologies include ligament cysts, tumors, and abnormal vascularization secondary to portal hypertension [3]. Additionally, the most recognized abnormalities of the falciform ligament are congenital pathologies including derivation and partial ligament defects [4]. It is well established that the arterial supply of the falciform ligament originates from a thin bundle branch of the right hepatic artery, which is anastomosed to the superficial inferior epigastric artery. In several cases, a bile duct may be localized within the ligament. Venous drainage flows directly into the paraumbilical vein and portal vein, while lymphatic drainage flows directly into the retroperitoneum [4]. Ying et al. [5] reported an arterial diameter of the falciform ligament of 1.8 ± 0.4 mm. Falciform ligament necrosis may likely develop, if the embolization of such a narrow artery or collateral venous flow fails due to vein thrombosis. Necrosis due to an occlusion of arterial supply is a rare primary falciform ligament disease. Review of the literature revealed limited data on falciform ligament necrosis or gangrenous disease with a similar clinical presentation. To the best of our knowledge, only ten cases with falciform ligament necrosis have been reported so far [6]. The majority of these patients are female patients with a mean age of above 60 years [7]. In our case, findings were also consistent with the literature data. In previous studies, patient's history and family's history were nonspecific. Half of the patients did not have a definite preoperative diagnosis [8]. In patients with acute abdomen, the decision for surgery was primarily based on clinical and physical examination findings, as well as laboratory test results and the presence of a tumor, as confirmed by imaging studies [9]. Similarly, the patient's history of this study was nonspecific for a definite preoperative medical pathology. The preliminary diagnoses delayed treatment of the duodenal perforation or mesenchymal tumor perforation. However, acute abdomen was accompanied by an intra-abdominal tumor-like mass in our case.

In recent years, the number of patients faced with the intraperitoneal fat tissue necrosis including the falciform ligament has been increasing parallel to the development in the field of radiology. The abdominal ultrasonography and computed tomography could provide important clues in the diagnosis of these patients. Coulier reported that the contrast-enhanced abdominal computed tomography is the gold standard for diagnosis of intraperitoneal fat necrosis as well as the follow-up of the disease [10]. Bacterial cultures of the blood and peritoneal fluid revealed no growth, which was consistent with previous cases. In contradistinction to the other cases in the literature, only Pans et al. reported* E. coli*,* Enterococcus*, and* Klebsiella* pneumonia in patient's culture with history of prosthetic mesh repair [9, 10]. No pathology such as* in situ* or distant cysts, abscess, and tumor was found, either. The diagnosis of primary falciform ligament necrosis was based on the absence of coagulation disorder or bacteremia.

The primary falciform ligament necrosis is often diagnosed during surgery. Coulier proposed that patients diagnosed with falciform ligament necrosis could be treated medically after excluding other disorders with a detailed history [10], whereas most writers still put forward to surgical treatment in patients with falciform ligament necrosis. In such patients, laparoscopy is another treatment option. Wider uses of diagnostic laparoscopy in acute abdomen have also picked up many such cases. But only a few cases of falciform ligament infarction have been reported [11]. Laparoscopy or laparotomy is indicated when the diagnosis is not clear or when there is no improvement on conservative measures. In our case, falciform ligament cysts, abscesses, and tumors such as pathologies and also any coagulation disorders or bacteremia were not found out. In accordance with these findings, our patient was diagnosed with primary falciform ligament necrosis.

In conclusion, falciform ligament necrosis is an extremely rare cause of acute abdomen. Surgeons should consider falciform ligament necrosis as a preliminary diagnosis if any ligament abnormality, tumor, intraligament air density, or the presence of reactionary fluid surrounding the ligament was detected through abdominal imaging studies. Surgery is the only treatment of choice. Laparoscopy or laparotomy can be performed according to the preference and experience of the surgeon as well as the overall condition of the patient.
